# Dissecting the transcriptional networks underlying breast cancer: NR4A1 reduces the migration of normal and breast cancer cell lines

**DOI:** 10.1186/bcr2610

**Published:** 2010-07-19

**Authors:** Annika N Alexopoulou, Maria Leao, Otavia L Caballero, Leonard Da Silva, Lynne Reid, Sunil R Lakhani, Andrew J Simpson, John F Marshall, A Munro Neville, Parmjit S Jat

**Affiliations:** 1 University of Oxford Branch, Ludwig Institute for Cancer Research, Old Road Campus, Off Roosevelt Drive, Oxford OX3 7DQ, UK; 2 Institute of Neurology, University College London, Queen Square, London WC1N 3BG, UK; 3 New York Branch at Memorial Sloan-Kettering Cancer Center, Ludwig Institute for Cancer Research, 1275 York Avenue, New York, NY 10021, USA; 4 Molecular and Cellular Pathology, University of Queensland Centre for Clinical Research and School of Medicine, The Royal Brisbane and Women's Hospital, Herston Road, Brisbane 4026, Australia; 5 Molecular and Cellular Pathology, University of Queensland Centre for Clinical Research, School of Medicine and Pathology, The Royal Brisbane and Women's Hospital, Herston Road, Brisbane 4026, Australia; 6 Invasion and Metastasis Laboratory, Tumour Biology Centre, Institute of Cancer, Queen Mary, University of London, Barts and the London Medical and Dental School, Charterhouse Square, London EC1 M 6BQ, UK

## Abstract

**Introduction:**

Breast cancer currently accounts for more than one-quarter of all female cancers and, despite the great progress in treatment observed in the past few years, the need for identification of new gene targets that can be used for diagnosis, prognosis and therapy is evident. A previous study identified the transcription factor NR4A1 as a gene upregulated in primary breast cancer compared with normal tissue by microarray analysis and sequencing technologies. The purpose of the study was to identify the role of NR4A1 in normal mammary epithelial and breast cancer cell biology.

**Methods:**

NR4A1 expression in breast tumours was assessed by semiquantitative and real-time PCR using RNA from normal and tumour samples or breast cancer cell lines. Immunohistochemistry on tissue microarrays was performed to check NR4A1 protein expression in breast tumours. MCF-10A and 226L normal mammary epithelial cells as well as the tumour lines PMC42, ZR-75-1 and MDA-MB-231 were transduced with full-length NR4A1, and the ability of NR4A1-overexpressing cells to migrate was tested using scratch wound or transwell migration assays. Proliferation was measured using the MTT and BrdU assays, while apoptosis was determined by the Annexin V assay. The ability of the cells to adhere to extracellular matrix was tested by adhesion assays and integrin cell surface expression was measured by flow cytometry. Activation of the FAK as well as ERK1/2 and PI3K pathways was checked by western blotting.

**Results:**

Breast tissue microarray analysis showed NR4A1 expression in primary tumours, which was reduced in higher grade and metastatic tumours. Ectopic expression of NR4A1 in MCF-10A, 226L, PMC42 and ZR-75-1 cells led to reduced ability of the cells to migrate, while no differences were observed in their proliferation and apoptotic index. NR4A1 expression altered the ability of the MCF-10A cells to adhere to the extracellular matrix and affected cell surface expression of integrins.

**Conclusions:**

NR4A1 acts as an antimigratory factor in two normal mammary epithelial and two breast cancer cell lines tested. It is therefore possible that NR4A1 acts as an antimigratory factor in breast tumours, and further studies should be conducted to understand the mechanisms involved.

## Introduction

Transcription factors are a family of proteins that regulate gene expression at different stages of embryonic development and are key to the establishment and maintenance of specific cell phenotypes. Consequently, their expression may have an important role in defining the neoplastic phenotype of an individual tumour. Dissecting transcriptional networks and targeting aberrantly expressed transcription factors has already become an important paradigm for cancer therapy, the oestrogen receptor being an important example.

Breast cancer is a clinically and structurally heterogeneous disease. The tumour itself consists of many different cell types, including normal and reactive stromal cells in addition to cancer cells [1,2]. The normal breast terminal duct-lobular unit is considered the origin of most cancers and consists of two morphologically recognisable cell types: epithelial cells on the inner luminal surface, surrounded by an outer layer of contractile myoepithelial (basal) cells. While typical breast cancers have been regarded traditionally as exhibiting characteristics akin to luminal epithelial cells, recent data have shown that some also exhibit, in part or whole, myoepithelial/basal features [3-5].

Gene expression profiling of RNA from solid heterogeneous breast tumours has enabled their classification into at least five different types [6,7] and gene signatures have been defined that are indicative of poor prognosis [8-10]. However, the precise nature of the RNA changes in the various types of cancer cells-even with prior laser microdissection capabilities - still remains elusive.

Our approach to ascertain the alterations present only in cancer cells has involved the use of immunomagnetic methods to separate malignant cells from other contaminating nonmalignant and stromal cell types within cancers, and also to separate the normal luminal and myoepithelial cells from fibroblasts, immune and endothelial cells within reduction mammoplasty material [11-13]. RNA extracted from purified luminal, myoepithelial and malignant cells from multiple donors was profiled using a multi-platform expression analysis, involving a combination of massively parallel signature sequencing and four different array-based genome-wide methods [12]. This has yielded what is probably the most comprehensive catalogue of genes whose levels are altered in breast cancer cells and whose expression can be annotated with respect to whether they represent luminal or myoepithelial type genes, free from the complexities due to the presence of normal and activated stromal cells present in solid tumour samples. This resulting differential tumour epithelial transcriptome (DTET) comprised 8,051 genes that were either upregulated or downregulated in the malignant epithelial cells. Ontological analysis has enabled these genes to be classified into different functional categories - the largest functional group of upregulated transcripts corresponded to genes associated with transcription and regulation of transcription, with potential to significantly influence the biology of a cancer cell by causing large-scale changes in gene expression [12].

In the present article we explore the role in mammary cell biology of one transcription factor, namely NR4A1, by its ectopic expression in normal mammary epithelial cells and breast cancer cell lines. NR4A1 expression reduces migration in both normal and tumour lines, as well as altering adhesion to the extracellular matrix (ECM) and integrin cell surface expression in MCF-10A cells, suggesting that NR4A1 may have an inhibitory role in relation to invasion/metastasis development.

## Materials and methods

### Materials

Antibodies against NR4A1 (clone P15 and M210) were obtained from Cell Signalling (Danvers, MA, USA) and Santa Cruz (Heidelberg, Germany). Antibodies against α_5 _integrin (P1D6) and α_6 _integrin (GOH3) were from Santa Cruz. Antibodies against α_1 _integrin (FB12), α_2 _integrin (P1E6), β_1 _integrin (P4C10), β4 integrin (3E1), αvβ_3 _integrin (LM609) and αvβ_6 _integrin (10D5) were obtained from Millipore (Watford, UK). The antibody against EMA (ICR2) was from Seralab (Leicestershire, UK), while the antibody against β_4 _integrin (A9) was from Santa Cruz. The Alexa-488 conjugated secondary antibodies were obtained from Invitrogen (Paisley, UK). Rabbit monoclonal or polyclonal antibodies against pERK1/2, total ERK1/2, pAKT, total AKT, pFAK (Y397) and total FAK were obtained from Cell Signalling. The anti-β-tubulin antibody, fibronectin (human plasma), vitronectin (bovine plasma), insulin, hydrocortisone, epidermal growth factor (EGF) and β-oestradiol were from Sigma-Aldrich (Dorset, UK). Collagen I (rat tail) was from BD Biosciences (Oxford, UK) and laminin I was from R & D (Abingdon, UK). DMEM/F12, DMEM, Leibovitz's medium and soybean trypsin inhibitor were from Invitrogen. Cholera toxin was from Biomol (Exeter, UK).

### Preparation of RNA from normal breast and tumours

Ten reduction mammoplasties were used to sort luminal epithelial cells by magnetic activated cell sorting (Miltenyi Biotec, Bisley, UK) as described previously [12]. Luminal epithelial cells were positively sorted using an antibody against the luminal marker EMA (clone ICR2) and were negatively sorted using an antibody against β_4 _integrin (clone A9).

The 226L normal mammary epithelial cell line was derived by immortalising sorted luminal cells with retroviruses that transduced SV40 large T antigen and the catalytic subunit of human telomerase [14].

Fifty-six primary grade 2 and grade 3 infiltrating ductal carcinomas were isolated as previously described [12]. RNA was extracted from 36 of the above tumour samples. RNAs were pooled for the first round of RT-PCR validation, while individual samples were used for the second round of RT-PCR validation.

Immunomagnetic cell sorting was performed for 20 out of the 56 solid tumours to remove desmoplastic fibroblasts. The tumour samples were negatively sorted using an antibody against fibroblast activation protein (clone F19) as previously described [12], and RNA was extracted resulting in the F19-negative RNA samples. A pool of these samples was used for the first round of RT-PCR validation, and individual samples were used for the second round.

A different set of F19-negative RNA samples was prepared and used for the real-time PCR experiments.

Informed consent was obtained from the patients. Samples were obtained from the Royal Marsden Hospital (London, UK) with approval from the appropriate Ethical Committee.

### Breast tissue microarray analysis

Formalin-fixed paraffin-embedded tumour blocks were retrieved from the archives of the Pathology of Queensland and the Wesley Hospital in Australia, from the Medical Faculty of Charles University in Plzen, Czech Republic, and from the Instituto Nacional do Cancer and Laboratorio Salomao & Zoppi in Brazil. The samples were anonymous and could not be tracked back to the patients. The study was approved by the local research ethics committees under project number UQ2005000785 and RBHW 2005/22.

One tissue microarray purely of lobular carcinomas and another two microarrays containing primary infiltrating ductal carcinomas and corresponding brain metastasis were built. None of these tumours relate to the ones used for RT-PCR and real-time PCR. Tissue microarrays were built using the tissue arrayer model MTAI (Beecher Instruments, Inc., Sun Prairie, WI, USA).

For immunohistochemistry, sections were cut at 4 μm and mounted on silane-coated slides. Immunohistochemistry was performed using the Envision dual-link system according to the manufacturer's (DAKO, Glostrup, Denmark) instructions. The polyclonal antibody to NR4A1 (clone M210) was used at a concentration of 1:100 and an incubation time of 60 minutes was employed, with antigen retrieval being achieved through the use of ethylenediamine tetraacetic acid (EDTA), pH 8.0, for 2 minutes at 105°C.

Positive (normal epithelial breast cells) and negative (exclusion of the primary antibody from the reaction) controls were included and the results were assessed by two pathologists under a double-headed microscope. Tumours were considered positive when > 10% of the tumour cells exhibited a positively stained cytoplasm. Nuclear staining was not observed.

### Cell culture

MCF-10A cells were obtained from ATCC-LGC Standards (Teddington, UK) and were cultured in DMEM/F12 medium supplemented with 5% horse serum, EGF (20 ng/ml), insulin (10 μg/ml), hydrocortisone (0.5 μg/ml) and cholera toxin (100 ng/ml). 226L cells were cultured in DMEM/F12 medium supplemented with 10% FCS, EGF (20 ng/ml), insulin (5 μg/ml), hydrocortisone (1 μg/ml) and cholera toxin (20 ng/ml). PMC42 cells [15,16] were cultured in the same medium as 226L cells in the absence of cholera toxin. ZR-75-1 cells were cultured in DMEM in the presence of 10% FCS and 10^-8 ^M β-oestradiol. MDA-MB-231 cells, a kind gift from Dr Tencho Tenev (The Institute of Cancer Research, London, UK) were cultured in DMEM with 10% FCS and 2 mM glutamine. Phoenix amphotropic cells and HEK293T cells (ATCC-LGC standards) were cultured in DMEM supplemented with 10% FCS and 2 mM glutamine.

### Plasmid constructs, retroviral and lentiviral infections

The full-length human NR4A1 ORF (Geneservice, Cambridge, UK) was cloned into the *Xho*I/*Not*I sites of the retroviral pLPCX vector (Clontech, Saint-Germain-en-Laye, France). For retroviral production, Phoenix amphotropic cells were transfected with pLPCX vector control or pLPCX-NR4A1, using FuGENE 6 (Roche, Mannheim, Germany). The full-length NR4A1 ORF was also cloned into the *Bam*HI/*Not*I sites of the pLEX lentiviral vector (Thermo Fisher Scientific, Huntsville, AL, USA). For lentiviral production, HEK293T cells were transfected with the pLEX or pLEX-NR4A1 following the protocol given by the UCL RNAi consortium (University College London, UK). MCF-10A cells were infected with the retroviral constructs, while all other lines were infected with the lentiviral ones.

For viral infections, viral supernatant was applied to the cells in the presence of 8 μg/ml polybrene (Sigma). To obtain stable expressing cell lines, infected cells were put under puromycin selection (1 μg/ml) 48 hours after infection.

### RNA extraction and RT-PCR

RNA was extracted using TRIzol reagent (Invitrogen) according to the manufacturer's instructions. RT was performed using SuperScript II (Invitrogen), and PCR was performed using GoTaq^® ^DNA polymerase (Promega, Southampton, UK).

### Real-time PCR

Normal tissue RNA preparations were purchased from Clontech Laboratories, Inc. (Palo Alto, CA, USA) and Ambion, Inc. (Austin, TX, USA). RT was performed using the Omniscript RT kit (Qiagen, Valencia, CA, USA) according to the manufacturer's instructions.

cDNA samples were run in duplicate for NR4A1 and for the reference gene within the same experiment using the Applied Biosystems apparatus 7500 Fast Real-Time PCR system and Taqman platform (Applied Biosystems, Foster City, CA, USA). HPRT1 was amplified as an internal reference gene. The PCR primers and probes were purchased from Applied Biosystems. Each sample was tested in two independent experiments. The values obtained for each sample from the two experiments were averaged and everything was compared with the normal breast.

### Western blot

Total protein was extracted using RIPA buffer (Sigma). Then 35 μg protein was electrophoresed on 10% Tris-glycine gels (Invitrogen) and transferred to nitrocellulose (GE Healthcare, Little Chalfont, UK). After blocking with 5% nonfat dry milk in Tris-buffered saline-Tween for 1 hour, the membranes were incubated with primary antibodies overnight at 4°C in 5% BSA Tris-buffered saline-Tween solution, washed and incubated with the appropriate horseradish peroxidase-conjugated secondary antibodies (GE Healthcare) for 1 hour at room temperature and visualised using ECL (GE Healthcare). Blots were washed, blocked and re-blotted with an anti-β-tubulin antibody, used as an internal loading control. For quantification of band intensity, the gel analysis function of ImageJ (National Institute of Health, USA) was used and the band intensity was standardised to β-tubulin. To determine statistical significance at each time point, the values obtained from different experiments for each cell line were compared using the paired Student's *t *test function of Microsoft Excel.

### Flow cytometry analysis of integrin expression

Cells were detached with trypsin/EDTA, washed and resuspended in flow cytometry buffer (0.5% BSA, 2 mM EDTA). Then 1 × 10^6 ^cells were stained with the appropriate primary antibody for 20 minutes at 4°C, and were washed and stained with secondary antibody solution for the same period of time. Dead cells were excluded with Topro-3 (100 nM; Invitrogen). Background fluorescence was measured using cells stained with secondary antibody only. For staining with the αvβ_6 _antibody, serum-free DMEM/F12 medium supplemented with 0.1% BSA was used throughout the procedure.

Data were collected using an LSRII flow cytometer (BD Biosciences) and results were analysed using FloJo software (Tree Star Inc., Ashland, Oregon, USA). The gate for positive cells was set at 1% of the secondary-only control cells with highest fluorescence. To compare fluorescence intensities, the median of the fluorescence intensity of secondary-only control cells was subtracted from the median fluorescence intensity of the samples.

### Scratch wound migration assays

MCF10A-NR4A1 cells or control cells were grown to confluency in 35 mm wells. Wounds were scratched using a 200 μl pipette tip and cells were washed three times with PBS. Assay medium was then added (DMEM/F12, 2% horse serum, 20 ng/ml EGF, insulin (10 μg/ml), hydrocortisone (0.5 μg/ml) and cholera toxin (100 ng/ml)). Triplicate wells were used per condition and three fields per well were photographed at each time point over a period of 24 hours. Scratch wound assays were performed for cells from three independent infections. For experiments where the MEK1/2 inhibitor U0126 was used, confluent monolayers were treated with the inhibitor 1 hour before wound initiation. Wounds were then scratched and cells were allowed to migrate in the assay medium supplemented with 10 μM inhibitor.

Wounds were visualised using a Nikon Eclipse TS100 microscope (Nikon, Kingston Upon Thames, UK), images were captured using a Coolpix 4500 camera (Nikon) and analysed by ImageJ software to calculate the distance covered by the migrating cells. For time-lapse microscopy, six-well plates with cells treated as above apart from the use of Leibovitz's medium instead of DMEM/F12 were fixed on the stage of a Carl Zeiss Axiovert 200 M microscope (Carl Zeiss Ltd, Hertfordshire, UK), and cells were maintained at 37°C in the absence of carbon dioxide. Images were captured using a Hamamatsu Orca C4742-80 12AG camera (Hamamatsu Photonics UK Ltd, Hertfordshire, UK), while image acquisition was controlled by Improvision Volocity versions 4.4 and 5.2 (PerkinElmer, Cambridge, UK), also used to create the QuickTime movies.

### Transwell migration and invasion assays

MCF-10A cells (5 × 10^4 ^cells) were plated in the top chambers of 8 μm pore transwells (BD Biosciences) in the full culture medium minus EGF and were allowed to migrate towards medium supplemented with 20 ng/ml EGF over a period of 24 hours. At the end of the assay, cells at the top chamber were removed and the cells at the bottom of the filter were fixed with 100% ethanol for 10 minutes and stained with 0.1% crystal violet solution for 30 minutes. The dye was eluted using 33% acetic acid, and crystal violet absorbance was measured at 590 nm. Quadruplicate wells were used per condition in each experiment. Transwell migration assays were performed six times in two independent infections. For all other lines, 5 × 10^4 ^to 1 × 10^5 ^cells were plated on the top of transwells in serum-free medium and were allowed to migrate either for 8 hours (226L and MDA-MB-231 cells) or 17 hours (PMC42 and ZR-75-1 cells) towards full medium. Experiments were repeated three times for each line.

For the invasion assays the same protocol as above was used with matrigel-coated transwells (BD Biosciences).

### Adhesion assays

Wells of a 96-well plate were coated with 2 μg/ml fibronectin, 4 μg/ml collagen type I, 8 μg/ml vitronectin, 8 μg/ml laminin I or 1% heat-inactivated BSA for 1 hour at room temperature. They were then blocked with 1% BSA for 2 hours at room temperature. Cells were detached using trypsin EDTA, which was inactivated with DMEM/F12 medium supplemented with soybean trypsin inhibitor (250 μg/ml) and 0.1 mg/ml BSA. Cells were resuspended in serum-free DMEM/F12 medium supplemented with 0.1 mg/ml BSA. Then 2 × 10^4 ^cells were plated in the fibronectin and collagen type I-coated wells, 4 × 10^4 ^cells were plated in the vitronectin-coated wells, and 6 × 10^4 ^cells were plated in the laminin I-coated wells and were incubated for 40 minutes in a 37°C incubator with a 5% carbon dioxide atmosphere. Unattached cells were removed with two washes of PBS and the adherent cells were fixed, stained with crystal violet and the absorbance was analysed as for the transwell migration assay. Six wells were used per condition in each experiment. Three or more experiments were performed on each matrix. Cells from two independent infections were used for the fibronectin experiments.

### MTT cell proliferation assay

The 3-(4,5-dimethylthiazol-2-yl)-2,5-diphenyltetrazolium bromide (MTT) cell proliferation assay (Invitrogen) was performed according to the manufacturer's instructions. Briefly, 2 × 10^3 ^to 6 × 10^3 ^cells were plated per well of a 96-well plate in full culture medium. To label the cells, MTT solution was added to the culture medium to a final concentration of 1.2 mM and incubation was continued for another 4 hours. Dimethylsulphoxide was then added and readings were taken on a plate reader at 570 nm with a reference wavelength of 690 nm. Readings for day 0 and day 1 were taken 24 hours and 48 hours after plating the cells, respectively.

### BrdU assay

Growing cells were incubated for 1 hour with Brdu (10 μM; Sigma) and the cells were trypsinised and fixed for at least 30 minutes on ice with 70% ice cold ethanol. The cells were then incubated with 2 M hydrochloric acid for 30 minutes, washed and double-stained with an anti-BrdU antibody (BD Biosciences) and propidium iodide. Data were collected using an LSRII flow cytometer and results were analysed using FloJo software.

### Apoptosis assay

Apoptosis was measured using FITC-conjugated Annexin V. Cells were grown in normal culture medium, trypsinised, washed twice in full medium and resuspended in 100 μl Annexin V binding buffer. Then 5 μl Annexin V (BD Biosciences) were added as well as propidium iodide to a final concentration of 5 μg/ml, and the cells were incubated at room temperature for 15 minutes in the dark. Annexin V binding buffer (400 μl) was then added and flow cytometry analysis was performed using an LSRII flow cytometer. Nonapoptotic cells were negative for both Annexin V and propidium iodide, early apoptotic cells were positive for Annexin V only, while cells positive for both Annexin V and propidium iodide were considered late apoptotic cells or already dead.

### Immunofluorescence

For immunofluorescence staining, cells were grown on glass coverslips or Lab-Tek permanox chamber slides (VWR, Lutterworth, UK). They were then fixed in a 3.5% paraformaldehyde solution at room temperature for 15 minutes, permeabilised with 0.1% Triton-X solution for 10 minutes, and washed and blocked in 0.1% BSA solution for 15 minutes at room temperature. Cells were stained with a rabbit polyclonal antibody against NR4A1 for 1 hour at room temperature and were washed three times with PBS. Secondary antibody solution was added for 1 hour at room temperature and the coverslips were then mounted. Images were visualised using a Zeiss Axioplan 2 microscope, captured using an AxioCam MRm camera, while image collection was controlled by AxioVision software (Carl Zeiss Ltd).

### Determination of EGF-mediated pathway activation

Confluent monolayers of control cells and MCF10A-NR4A1 cells were grown in normal culture medium in the absence of EGF and in the presence of 2% horse serum overnight. Twelve wounds were scratched per well of a six-well plate, and assay medium supplemented with 20 ng/ml EGF was added. Protein lysates were collected at different time points and were analysed for the expression of phospho-ERK1/2 and total ERK1/2, AKT and FAK by western blotting.

### Statistical analysis

In each experiment the mean and standard deviation for multiple wells was calculated using Excel. Statistical significance (*P *< 0.05) was determined using the unpaired two-tailed Student's *t *test.

To determine the mean values obtained from repeats of experiments, the mean value obtained for control cells in each experiment was set as 100%. The percentage of mean difference ± standard error (SE) between control cells and cells ectopically expressing NR4A1, as well as the statistical significance, were calculated by univariate analysis of variance using the SPSS 15.0 software (SPSS, Chicago, IL, USA). Yates' chi-square test was used for the clinical breast cancer/metastasis samples.

## Results

### RT-PCR analysis of differentially expressed transcription factors identified in the differential tumour epithelial transcriptome

The DTET comprises 8,051 transcripts that show a significant difference in abundance between normal luminal epithelial cells and primary malignant epithelial cells, and 640 of these transcripts belong to the family of transcription factors [12].

Of the 640 differentially expressed transcription factors that were identified as differential, 275 were chosen for validation (Additional file 1). To confirm the differential expression of these candidates, semiquantitative RT-PCR analysis was performed using a pool of RNA from normal luminal epithelial cells, RNA prepared from a minimally immortalised normal epithelial cell line, a pool of RNA from F19-negative (tumour cells negatively sorted with the F19 antibody against fibroblast activation protein) primary breast tumour preparations and a pool of RNA extracted from solid tumours. Twenty-four transcription factor genes were clearly identified as differentially expressed (Table 1 and Additional file 2).

**Table 1:** Genes deregulated in breast cancer according to the first round of RT-PCR validation

Hugo name	Reference sequence	Regulation in tumours
AEBP1	NM_001129	Upregulated
BCL6B	NM_181844	Upregulated
BNC1	NM_001717	Downregulated
ELF5	NM_001422, NM_198381	Upregulated
ENPP2	NM_006209	Upregulated
HCLS1	NM_005335	Upregulated
HHEX	NM_002729	Upregulated
HOXA3	NM_153631, NM_153632, NM_030661	Downregulated
IRF4	NM_002460	Upregulated
KLF2	NM_016270	Upregulated
LASS4	NM_024552	Upregulated
LBP-9	NM_014553	Downregulated
LDB2	NM_001290	Upregulated
MEOX1	NM_004527/NM_013999	Upregulated
MTA1	NM_004689	Upregulated
NR4A1	NM_002135/NM_173157	Upregulated
PBX1	NM_002585	Upregulated
PHF13	NM_153812	Upregulated
ZMYND8	NM_012408, NM_183047, NM_183048	Upregulated
SPDEF	NM_012391	Upregulated
TCF8	NM_030751	Upregulated
TRIM29	NM_012101	Downregulated
ZNF114	NM_153608	Downregulated
ZNF277	NM_02994	Downregulated

**Figure 1:**
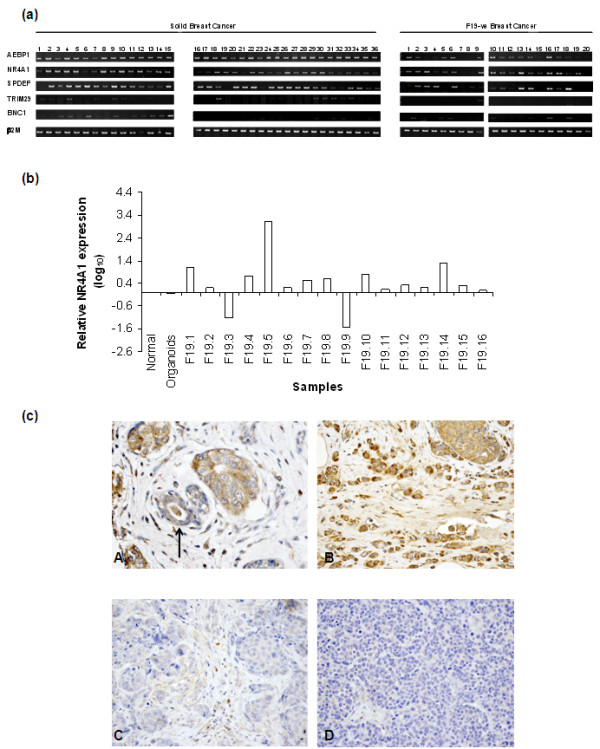
**RT-PCR validation of differential expression of transcription factors identified in the differential tumour epithelial transcriptome**. **(a) **Validation of 275 transcription factors that had been identified in the differential tumour epithelial transcriptome (DTET) to be deregulated in breast tumours was performed by RT-PCR. The first round of validation, where pools of normal and tumour RNAs were used, confirmed 24 genes to be deregulated in breast cancer. These 24 genes underwent a second round of RT-PCR validation in which RNAs from individual solid and F19-negative breast cancer samples were used. Genes deregulated in breast cancers were considered those showing differential expression in 50% or more of the F19-negative tumours compared with normal breast. The genes shown are the five transcription factors identified by the DTET and confirmed by two rounds of RT-PCR validation to be deregulated in tumours. β_2_-microglobulin (β2 M) was used as a loading control. **(b) **Real-time PCR was performed for NR4A1 expression using RNA from normal breast, breast organoids and F19-negative tumours. Nine out of the 16 F19-negative tumours tested showed twofold or higher NR4A1 expression compared with normal breast (F19.1, F19.4, F19.5, F19.7, F19.8, F19.10, F19.12, F19.14 and F19.15). **(c) **Immunohistochemical staining of breast tissue microarrays. (A) Normal lobule (arrow) with weak staining in luminal cells and adjacent island of ductal carcinoma with positive staining. (B) Positive staining in grade 2 invasive and *in situ *lobular carcinoma. Negative staining in (C) primary high-grade ductal carcinoma and (D) its matched brain metastasis.

These 24 transcription factors were further analysed by semiquantitative RT-PCR using a large panel of RNAs extracted from normal cells, breast cancer cell lines, solid breast tumours and F19-negative breast cancer cells. While five of these genes were confirmed to be deregulated in breast cancer (Figure 1a), our attention became focused on the orphan nuclear receptor NR4A1 - which was further confirmed to be upregulated twofold or more compared with normal breast in 9 out of the 16 F19-negative samples tested by real-time PCR (Figure 1b).

### NR4A1 protein is expressed in breast tumours

To determine NR4A1 protein expression in breast tumours, tissue microarrays of lobular and infiltrating ductal carcinomas were used for immunohistochemistry.

In the normal attached breast tissue, NR4A1 cytoplasmic staining was weak. Analysis of the pathological data showed more prominent cytoplasmic staining levels in breast cancers and a negative association of NR4A1 with increasing degrees of histological grade among the infiltrating duct carcinomas, and hence invasive potential (Figure 1c and Table 2). Only 14 out of 48 grade 3 infiltrating ductal carcinomas were positive for NR4A1, compared with 23 out of 31 grade 1/2 infiltrating ductal carcinomas (chi-squared test *P *= 0.0002; Table 2). In some instances it was possible to compare the primary tumours with their metastasis. Of the series of grade 3 infiltrating carcinomas, 31 went on to develop 41 brain metastasis. While the primary tumours were positive in 16% (5/31) of cases, only 2% (1/41) of the metastasis were positive for NR4A1 - further confirming its negative association with higher degrees of invasive potential (Figure 1c and Table 2). The lobular carcinomas in the present series all fell into the grade 2 category and also showed a trend for increase of expression (chi-squared test *P *= 0.12; Table 2) of NR4A1 compared with grade 3 primary infiltrating ductal carcinomas.

**Table 2:** Breakdown of the analysis of NR4A1 status in 160 breast carcinomas

Tumour status	Histological type of carcinoma	NR4A1 status	Grade	Proportion of positive cases	Range of positivity	
					
					10 to 50%	> 50%
Primary	Lobular	Present	2	36/81 (44%)	2/81 (2%)	34/81 (42%)
Primary	IDC	Present	1 and 2	23/31 (74%)	3/31 (9%)	20/31 (64%)
Primary	IDC	Present	3	14/48 (29%)	0	14/48 (29%)
Metastatic	IDC	Present	3	1/41 (2%)	0	1/41 (2%)

Table depicts grade, histological type, proportion and range of positivity. Samples with > 10% positive cells were considered positive. IDC, infiltrating duct carcinoma.

### Ectopic expression of NR4A1 in MCF-10A cells

**Figure 2:**
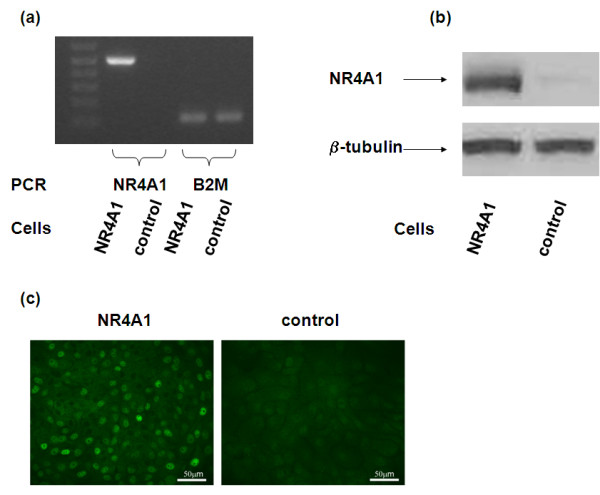
**NR4A1 overexpression in mammary epithelial cells**. MCF-10A cells were infected either with a retroviral construct containing the NR4A1 cDNA or with an empty vector control. Overexpression was confirmed at the mRNA level by **(a) **RT-PCR, and at the protein level by **(b) **western blot and **(c) **immunofluorescence.

Since our data show that NR4A1 expression is increased in primary tumours compared with normal breast (Figure 1) we examined its biological role in breast biology. NR4A1 was ectopically expressed in the immortalised mammary epithelial cell line MCF-10A using retroviral vectors. Overexpression of NR4A1 was confirmed at the mRNA level by RT-PCR (Figure 2a) and at the protein level by western blotting (Figure 2b) and immunofluorescence (Figure 2c). NR4A1 was not detected in the parental MCF-10A cells but it was readily detected upon ectopic expression and localised to the nucleus.

### NR4A1 decreases the migratory potential of MCF-10A cells

To determine whether NR4A1 has an effect on MCF-10A cell migration, control cells and MCF10A-NR4A1 cells were used in scratch wound and transwell migration assays.

**Figure 3:**
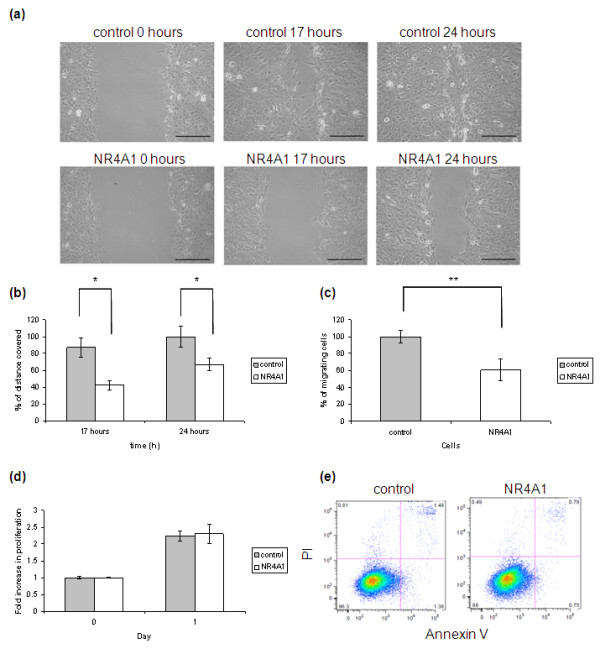
**NR4A1 reduces the migratory ability of MCF-10A cells**. **(a) **A wound was scratched in a confluent monolayer of control and MCF10A-NR4A1 cells. The edge of the wound was monitored over 24 hours and pictures of the same fields were taken at 0, 17 and 24 hours. Scale bars: 300 μm. **(b) **Percentage migration. **(c) **To confirm the migration phenotype, cells were plated on the top chamber of transwells in the absence of EGF and were allowed to migrate overnight towards medium supplemented with EGF present in the lower chamber. **(d) **To compare the proliferation rate of control and MCF10A-NR4A1 cells, the MTT assay was performed. **(e) **The Annexin V assay was performed to detect apoptosis in the two cell lines. Graphs show the mean ± standard deviation of representative experiments out of at least *n *= 3. *P < 0.001, **P < 0.005. PI, propidium iodide.

Preliminary experiments of scratch wound assays showed that EGF was required for MCF-10A cells to close the wound, and that in its absence wounds remained open even after 5 days (data not shown). The assays were therefore performed in the presence of EGF, and pictures of the same fields were taken at 0, 17 and 24 hours (Figure 3a). While control cells had almost closed the wounds at 24 hours, the wounds of the MCF10A-NR4A1 cells were still open at this time. MCF10A-NR4A1 cells covered 49 ± 1.5% and 58 ± 1.8% of the distance covered by the control cells at 17 hours and 24 hours, respectively (mean ± SE, *n *= 3, *P *< 0.001; Figure 3b). Immunofluorescence microscopy of cells fixed 17 hours after initiation of the scratch wound assay shows that NR4A1 is expressed by the MCF10A-NR4A1 cells at the edge of the wound (Additional file 3). Time-lapse microscopy was performed over a period of 24 hours, confirming that wound closure occurred due to cell spreading and migration and not due to cell proliferation (Additional file 4 and 5). To confirm the migration phenotype, control cells and MCF10A-NR4A1 cells were subjected to transwell assays and allowed to migrate overnight towards EGF-supplemented medium in the bottom chamber. This assay showed a 32 ± 4.2% reduction (mean ± SE, *n *= 6, *P *< 0.001) in the migration of the MCF10A-NR4A1 cells compared with control cells (Figure 3c), confirming the results obtained by the scratch wound assay.

The MTT cell viability assay was also performed to test whether a difference in cell proliferation could account for the differences in cell migration observed (Figure 3d). Importantly, no difference in the proliferation rate of the two cell lines was observed by this MTT assay. Furthermore, flow cytometry analysis of cells incubated with BrdU for 1 hour and stained with an anti-BrdU antibody confirmed that the rate of BrdU incorporation is similar between control cells and MCF10A-NR4A1 cells (Table 3 and Additional file 6). Similarly, no difference in the apoptotic index of control cells and NR4A1-expressing cells was observed according to the Annexin V apoptosis assay (Figure 3e and Table 4).

**Table 3:** Summary of data from flow cytometry analysis of BrdU incorporation in normal and tumour lines

Cells	% BrdU incorporation		Significant*
		
	Control cells	NR4A1 cells	
MCF-10A (*n *= 3)	22 ± 1.7	20 ± 0.1	No
PMC42 (*n *= 3)	50 ± 1	42 ± 3.6	No
ZR-75-1 (*n *= 2)	24	21	N/A
MDA-MB-231 (*n *= 3)	33 ± 5	33 ± 3.5	No
226L (*n *= 3)	18 ± 2.7	16 ± 4.6	No

Data presented as mean ± standard error. *P < 0.05 by paired Student's *t *test.

**Table 4:** Results summary for the Annexin V assay performed in normal and breast cancer cell lines

Cells	Annexin V positive cells (%)	Annexin V and propidium iodide positive cells (%)	Total Annexin V positive cells (%)
MCF10A-cotntrol (*n *= 2)	1.7	1.6	3.3
MCF10A-NR4A1 (*n *= 2)	1	1	2
PMC42-control (*n *= 3)	3 ± 1.1	8 ± 1.2	11 ± 2
PMC42-NR4A1 (*n *= 3)	1.5 ± 0.5	7 ± 0.8	8.5 ± 0.7
ZR75-1-control (*n *= 3)	1.8 ± 0.5	11 ± 1.2	12.8 ± 1.7
ZR75-1-NR4A1 (*n *= 3)	1.6 ± 0.6	13 ± 2.8	14.6 ± 2.3
MDA-MB-231-control (*n *= 3)	2.3 ± 0.6	10 ± 2	12.3 ± 2.5
MDA-MB-231-NR4A1 (*n *= 3)	2 ± 0.6	8 ± 1	10 ± 1.3
226L-control (*n *= 3)	6 ± 0.6	14 ± 1	20 ± 0.3
226L-NR4A1 (*n *= *n *= 3)	3.6 ± 1	10 ± 1.7	13.6 ± 1.5

Data presented as mean ± standard error.

### NR4A1 reduces migration in normal mammary epithelial cells and breast cancer cell lines

**Figure 4:**
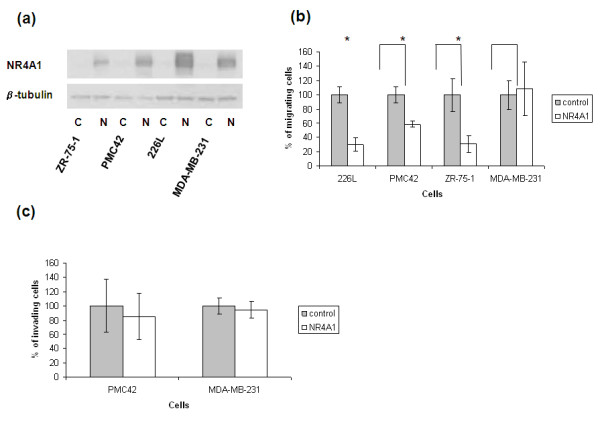
**NR4A1 reduces the migratory ability of breast cancer lines**. NR4A1 was ectopically expressed in three breast cancer lines (ZR-75-1, PMC42 and MDA-MB-231) and one normal mammary epithelial cell line (226L). **(a) **Ectopic expression was confirmed at the protein level by western blot. **(b) **The cell lines were then used for transwell migration assays where cells were allowed to migrate for 8 hours (226L and MDA-MB-231) or overnight (PMC42 and ZR-75-1) towards full culture medium. In all lines apart from MDA-MB-231, NR4A1 reduced the migratory ability of the cells. **(c) **The invasive lines PMC42 and MDA-MB-231 were used in transwell invasion assays. Cells were allowed to invade through the matrigel-coated transwells for 8 hours (MDA-MB-231) or overnight (PMC42). NR4A1 expression had no effect on the invasion of the tumour lines. Graphs show the mean ± standard deviation of one representative experiment out of *n *= 3. *P < 0.001. C, control; N, NR4A1.

To determine whether the ability of NR4A1 to reduce migration is specific to the MCF-10A cells, another normal mammary epithelial cell line (226L) ectopically expressing NR4A1 (Figure 4a) was used in transwell migration assays. Similarly to the MCF10A-NR4A1 cells, 226L-NR4A1 cells showed reduced migration since only 42 ± 7.5% (mean ± SE, *n *= 3, *P *< 0.001) of these cells migrated compared with controls (Figure 4b). To determine whether NR4A1 can reduce migration in tumour cells, the breast tumour cell lines PMC42, ZR-75-1 and MDA-MB-231 ectopically expressing NR4A1 (Figure 4a) were used in transwell migration assays. NR4A1 reduced tumour cell migration, since 61 ± 2.1% (mean ± SE, *n *= 3, *P *< 0.001) and 44 ± 5% (mean ± SE, *n *= 3, *P *< 0.001) of PMC42-NR4A1 cells and ZR75-1-NR4A1 cells, respectively, migrated compared with control cells. No effect on the migration of the MDA-MB-231 cell line was observed (Figure 4b).

Since the levels of NR4A1 inversely correlate with tumour grade and invasiveness (Figure 1c), it was important to determine whether NR4A1 also has the ability to reduce tumour cell invasion. To determine whether NR4A1 affects this process, the invasive lines PMC42 and MDA-MB-231 were used in transwell invasion assays. NR4A1 had no effect on the invasion of either cell line through matrigel (Figure 4c).

NR4A1 has no effect on the proliferation rate or apoptosis index of any of the lines tested, as determined by BrdU incorporation and the Annexin V apoptosis assay, respectively (Tables 3 and 4, and Additional file 6 and 7).

### NR4A1 alters the ability of MCF-10A cells to adhere to the extracellular matrix and integrin cell surface expression

**Figure 5:**
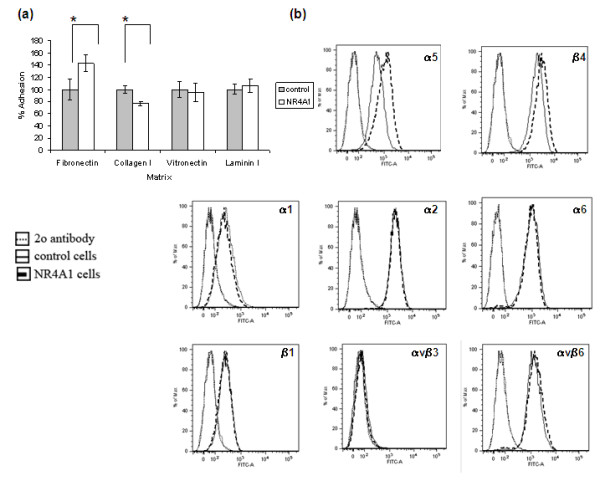
**Effect of NR4A1 on cell adhesion and integrin expression**. **(a) **Control or MCF10A-NR4A1 cells were plated on wells coated with fibronectin, collagen type I, vitronectin or laminin I. Attached cells were fixed and stained with crystal violet. MCF10A-NR4A1 cells show increased adhesion to fibronectin and decreased adhesion to collagen type I compared with control cells, while both cell lines show similar adhesion levels to vitronectin and laminin I (mean ± standard deviation of six wells, *P < 0.001). **(b) **MCF10A-NR4A1 cells or control cells were analysed by flow cytometry for the cell surface expression of the integrins depicted. MCF10A-NR4A1 cells have increased levels of α_5 _and β_4 _integrins.

Since cell adhesion and migration are two processes linked to each other, the ability of NR4A1 cells to adhere to the ECM was tested. MCF10A-NR4A1 cells showed increased adhesion to fibronectin (23 ± 4%, mean ± SE, *n *= 5, *P *< 0.001) and decreased adhesion to collagen type I (30 ± 3%, mean ± SE, *n *= 3, *P *< 0.001), while both control cells and MCF10A-NR4A1 cells adhered to vitronectin and laminin I at similar levels (Figure 5a).

Since the primary receptors for cell interaction with the ECM are integrins, flow cytometry analysis was performed to test whether the differences in the adhesion profile of the cells would be reflected in their cell surface integrin expression. Flow cytometry analysis for the expression of cell surface integrins showed that MCF10A-NR4A1 cells had an average of 61 ± 4.8% (mean ± SE, *n *= 6, *P *< 0.001) higher expression of α_5 _integrin and 73 ± 7.8% higher expression of β_4 _integrin (mean ± SE, *n *= 5, *P *< 0.001) compared with control cells (Figure 5b). Since α_5 _integrin is the alpha subunit of the α_5_β_1 _fibronectin receptor, increased levels of this integrin in the MCF10A-NR4A1 cells may be responsible for their increased adhesion to fibronectin (Figure 5a). Furthermore, no differences in the levels of αvβ_6 _integrin, another receptor for fibronectin, were observed. Although reduced adhesion to collagen type I was observed (Figure 5a), the expression of integrins α_1 _and α_2 _that form the collagen receptors α_1_β_1 _and α_2_β_1 _was the same between the two cell lines. Despite the fact that β_4 _integrin forms one of the main laminin receptors α_6_β_4_, the increased levels of the β_4 _subunit did not result in increased adhesion to laminin I. Finally, no differences in the levels of β_1_, αvβ_3 _and α_6 _integrins were observed (Figure 5b).

### NR4A1 expression affects the ERK1/2 pathway

Initial experiments (data not shown) showed that EGF is necessary for MCF-10A cell migration in the scratch wound assay. Two of the main pathways activated by EGF binding to the EGF receptor are the mitogen-activated protein kinase (MAPK) pathway and the phosphoinositide 3-kinase (PI3K) pathway [17]; both pathways have been shown to be involved in EGF-mediated epithelial cell migration [18-20].

**Figure 6:**
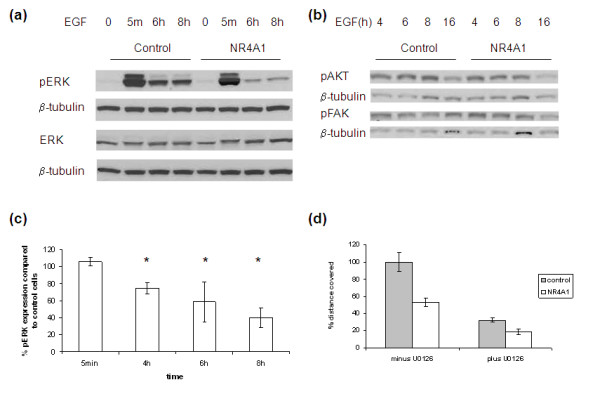
**NR4A1 affects the levels of active ERK1/2 during migration**. Confluent monolayers of control cells and MCF10A-NR4A1 cells were grown in normal culture medium in the absence of epidermal growth factor **(**EGF) and in the presence of 2% horse serum overnight. Twelve wounds were scratched per well and 20 ng/ml EGF was added for the time indicated. Protein lysates were analysed for the expression of **(a) **phospho-ERK1/2 and **(b) **phosphor-AKT and phospho-FAK. **(c) **Summary of results for active ERK1/2 from three or more experiments (mean ± standard deviation, *P < 0.05). **(d) **Confluent monolayers of control and MCF10A-NR4A1 cells were treated for 1 hour with 10 μM U0126 inhibitor. Wounds were then scratched and the cells were allowed to migrate for 17 hours in the presence of the inhibitor in EGF-supplemented medium. Pictures of the same fields were taken at 0 and 17 hours. A representative experiment is shown out of three performed.

To test whether NR4A1 affects the ERK/MAPK pathway, EGF-starved cells were treated with EGF and protein lysates were collected at different time points and tested for the expression of activated ERK1/2 (Figure 6a, c). Although the initial activation of ERK1/2 was the same in both control and MCF10A-NR4A1 cells (5 minutes), the NR4A1-expressing cells had lower levels of active ERK1/2 at later time points (4 to 10 hours). The importance of the ERK/MAPK pathway in MCF-10A cell migration was confirmed by repeating the scratch wound assay in the presence of a MEK1/2 inhibitor (U0126). The inhibitor reduced the migration of both control cells and MCF10A-NR4A1 cells (Figure 6d). On the other hand, the PI3K pathway did not seem to be affected as the levels of phospho-AKT were similar in both cell lines (Figure 6b). Finally, no differences in the levels of phospho-FAK were observed (Figure 6b).

## Discussion

Breast cancer is the most common cancer in European women, accounting for more than one-quarter of all female cancers. Despite the great progress observed in treating hormone-sensitive and ErbB2-overexpressing breast tumours, the need for further therapeutic targets has been recently emphasised [21]. Hence, there remains a need for the identification of new genes that can be used in diagnosis, prognosis and therapy.

In our study, NR4A1 was first identified in the DTET as one of the transcription factors upregulated in primary breast tumours [12]. Validation of these results by semiquantitative and real-time PCR confirmed the increased expression of NR4A1 in tumour cells (Figure 1a, b). Immunohistochemical analysis showed that NR4A1 protein is indeed expressed in tumours but only weakly in normal cells, and interestingly that its expression is downregulated in higher grade and metastatic tumours (Figure 1c and Table 2). These data prompted us to study the role of NR4A1 in mammary epithelial cell biology.

NR4A1 belongs to the NR4A family of nuclear receptors that also includes Nurr1 (NR4A2) and NOR-1 (NR4A3). No physiological ligand has been reported for the NR4A nuclear receptors, which are therefore classified as orphan receptors. NR4A family members are stress/immediate early response genes that can be induced by several signals such as stress, inflammatory cytokines, phorbol esters, peptide hormones, growth factors and neurotransmitters [22-27]. They play a role in the central nervous system, steroidogenesis, inflammation, cell survival and cell death [28-32].

NR4A1 is considered a mediator/regulator of cell survival and apoptosis in tumours. Several apoptotic agents - including etoposide, phorbol esters, calcium ionophore, retinoids and butyrate - induce the levels of NR4A1 and lead to its translocation from the nucleus to mitochondria, where it interacts with Bcl-2 and converts the latter into an inducer of apoptosis. NR4A1 has been shown to mediate apoptosis in several tumour cell lines, including prostate cancer cells, colon cancer cells, breast cancer cells and melanoma cells [29,33-36]. On the other hand, ectopic expression of NR4A1 promotes cell cycle progression in lung cancer cells, showing a requirement for its DNA-binding domain and transactivation [25], while it also mediates the mitogenic effects of vascular endothelial growth factor A, and NR4A1 null mice injected with melanoma cells show reduced tumour formation and angiogenesis [37].

The above studies suggest that NR4A1 plays distinct roles depending on the cell context and signal stimulation, and suggest that modulation of NR4A1 activity may be used as an anticancer therapy. Interestingly, knockout mice that are null for both NR4A1 and NOR1 develop acute myeloid leukaemia due to abnormal myeloid cell proliferation and apoptosis. Both transcripts have been shown in humans to be downregulated in patients with acute myeloid leukaemia [38]. While NR4A1 is expressed in melanoma tissues, its expression appears to decline in metastatic melanomas [36]. Finally, analysis of metastatic adenocarcinomas of diverse origin and comparison with primary adenocarcinomas of the same tumour spectrum identified NR4A1 as part of a 17-gene signature associated with metastasis as one of the downregulated genes [8]. The data from the present study are in agreement with the above studies, since they show NR4A1 cytoplasmic expression in primary tumours that is reduced in higher grade and metastatic tumours (Figure 1c and Table 2). Since cytoplasmic NR4A1 has been shown to mediate apoptosis in several cancer cell lines, its reduced expression in higher grade and metastatic tumours may possibly contribute to the survival of such tumour cells.

The above results prompted us to study the role of NR4A1 expression in mammary epithelial cells. For this purpose, the normal mammary epithelial cell line MCF-10A was infected with a retroviral construct containing the NR4A1 cDNA, and ectopic expression was confirmed both at the mRNA and protein levels (Figure 2). Interestingly, ectopically expressed NR4A1 was present in the nucleus and not in the cytoplasm of MCF10A-NR4A1 cells grown under normal culture conditions (Figure 2c). During characterisation of the MCF10A-NR4A1 cells, data obtained from both scratch wound and transwell migration assays showed that NR4A1 reduces the migratory ability of MCF-10A cells (Figure 3a to 3c). As NR4A1 can act both as a prosurvival and a proapoptotic agent depending on the cell context and stimulus, it was important to confirm that the results obtained regarding the cell migration phenotype were not due to differences in the proliferation or apoptosis of the two cell lines. Indeed the data from the MTT cell proliferation assay that measures mitochondrial activity showed no differences in cell viability and proliferation between control and MCF10A-NR4A1 cells (Figure 3d). Similar results were obtained by the BrdU incorporation assay (Table 3 and Additional file 6). Furthermore, no differences in apoptosis were observed according to the Annexin V staining (Figure 3e and Table 4). Time-lapse microscopy also confirmed that both cell lines migrated through spreading and not through proliferation (Additional file 4 and 5).

To test whether the ability of NR4A1 to reduce migration is specific to MCF-10A cells, another normal mammary epithelial cell line ectopically expressing NR4A1 (226L cell line, prepared in our laboratory) was used for transwell migration assays. The results obtained showed that NR4A1 also reduces migration in the 226L cells and therefore that its effect in not specific to the MCF-10A cells. As NR4A1 levels decline in higher grade tumours, it was hypothesised that NR4A1 may have an adverse effect on the migratory ability of tumour cells. Three breast cancer lines ectopically expressing NR4A1 (PMC42, ZR-75-1 and MDA-MB-231) were used to perform transwell migration assays. NR4A1 was able to reduce the migration in two out of the three lines tested (PMC42 and ZR-75-1; Figure 4b). As several studies have previously suggested that NR4A1 induces tumour cell apoptosis [29,33-36], the Annexin V assay was performed - showing that, in the case of the tumour lines tested, NR4A1 did not affect cell apoptosis (Table 4 and Additional file 7). The downregulation of NR4A1 in breast cancers of higher grade is therefore possibly due to its adverse effect on the migration of the tumour cells. As NR4A1 is downregulated in higher grade, more invasive tumours, it was also possible that it may have an effect on the invasive ability of tumour cells. However, no difference in the invasion of PMC42 and MDA-MB-231 cells through matrigel was observed (Figure 4c).

Cell adhesion to the ECM is important for the organisation and function of epithelial tissues. The main receptors for cell-ECM interaction are integrins, while heparan sulphate proteoglycans are also involved in this process [39]. Cell adhesion and migration are two processes linked to each other. Since NR4A1 was found to alter the migratory ability of MCF-10A cells, it was hypothesised that it would also alter their ability to adhere to ECM. Our results showed that NR4A1 enhances the adhesion of MCF-10A cells to fibronectin and reduces their adhesion to collagen type I, while it does not affect adhesion to vitronectin or laminin I (Figure 5a). Integrin expression profiling showed that MCF-10A cells have higher levels of α_5 _integrin, which along with the β_1 _subunit forms the α_5_β_1 _integrin receptor, one of the fibronectin receptors, as well as higher levels of β_4 _integrin (Figure 5b). As no difference in the levels of the αvβ_6 _fibronectin receptor was observed, this supports the hypothesis that increased adhesion to fibronectin is possibly through the α_5_β_1 _receptor. Interestingly, no differences in the expression levels of the α_1 _and α_2 _integrins that form the collagen receptors α_1_β_1 _and α_2_β_1 _were observed, despite the reduction in the adhesion of MCF10A-NR4A1 cells to collagen type I. It is therefore possible that the reduction in collagen adhesion in the latter cells is due to reduced levels of active integrins on the cell surface and not due to differences in expression levels. No differences in the ability of the cells to adhere to laminin I were observed, despite the increase in the levels of β_4 _integrin that forms one of the major receptors (α_6_β_4_) for adhesion to this ECM. This therefore suggests that, in the case of the MCF-10A cells, adhesion to laminin I occurs through a different integrin pair.

In cell migration, although the formation of integrin-mediated adhesions is required to create traction for cells to move, too strong adhesions or their reduced turnover inhibit migration. The reduced migration of MCF10A-NR4A1 cells may therefore be due to increased integrin levels. Our preliminary data using blocking antibodies against α_5 _or β_4 _integrins, however, do not support this hypothesis. The importance of β_4 _integrin in promoting breast cancer cell invasion and survival has previously been demonstrated [40]. Since NR4A1 appears to have an adverse effect on tumour cells, further studies will be conducted to determine whether NR4A1 actually regulates β_4 _integrin expression in breast tumour cells.

EGF induces robust migration in MCF-10A cells (our results; see also [18]) and the ERK/MAPK pathway has been shown to be involved in the process [18]. Our data showed that the initial EGF-mediated activation of the ERK/MAPK pathway is similar in both cell lines. The levels of active ERK1/2, however, appear to decline faster in the MCF10A-NR4A1 cells compared with the control ones (Figure 6a, c). Inhibition of the ERK/MAPK pathway resulted in reducing the migration of both control cells and MCF10A-NR4A1 cells (Figure 6d). The reduced levels of active ERK1/2 may therefore contribute towards the migratory phenotype of the MCF10A-NR4A1 cells.

Our data propose that nuclear NR4A1 may play a protective role against breast cancer metastasis as it acts to inhibit both normal and tumour cell migration. Our immunohistochemical data (Figure 1c and Table 2) also suggest that NR4A1 may play a protective role against metastasis, although the expression in this case was cytoplasmic. Higher grade 3 breast cancers (that is, those with a potentially poorer prognosis and the greater likelihood of metastasis in due course) are less likely to contain demonstrable levels of NR4A1. Breast cancer of all grades possesses the potential to spread and metastasise. This spread is less common with grade 2 lesions than those of grade 3, but it does occur. There is thus a continuum of invasive potential not indicated solely by the histological features. These tumours within each grade that possess demonstrable NR4A1 may therefore be those that are less likely to spread. Further studies with detailed follow-up and in comparison with other known prognostic factors will be needed to prove or disprove such a hypothesis.

## Conclusions

In summary, our study has shown that NR4A1 expression is reduced in metastatic breast tumours. NR4A1 has the ability to reduce the migration of breast tumour cells as well as the ability to reduce migration, to alter the adhesion to ECM and integrin cell surface expression of normal mammary epithelial cells. Further studies are being conducted to delineate the pathways involved in the phenotype observed.

## Abbreviations

BSA: bovine serum albumin; DMEM: Dulbecco's modified Eagle's medium; DTET: differential tumour epithelial transcriptome; ECM: extracellular matrix; EDTA: ethylenediamine tetraacetic acid; EGF: epidermal growth factor; ERK: extracellular signal-regulated kinase; FCS: foetal calf serum; MAPK: mitogen-activated protein kinase; MTT: 3-(4,5-dimethylthiazol-2-yl)-2,5-diphenyltetrazolium bromide; ORF: open reading frame; PBS: phosphate-buffered saline; PCR: polymerase chain reaction; PI3K: phosphoinositide 3-kinase; RT: reverse transcription; SE: standard error.

## Competing interests

The authors declare that they have no competing interests.

## Authors' contributions

ANA performed the infections, RT-PCR for NR4A1 ectopic expression, western blot assays, immunofluorescence, scratch wound assays, transwell migration and invasion assays, proliferation and apoptosis assays, adhesion assays, flow cytometry for integrin expression and tested the activation of FAK, as well as of the ERK1/2 and PI3K pathways, and drafted the manuscript. ML performed the RT-PCRs to confirm the deregulation of the transcription factors identified in the DTET. OLC performed the real-time PCR. LDS, LR and SRL performed the immunohistochemistry of tissue microarrays. AJGS contributed to the conception of the project. JFM has contributed to the supervision of the project and interpretation of results. AMN and PSJ have contributed to the supervision of the project and drafting of the manuscript.

## Acknowledgements

The present work was conducted as part of the Hilton-Ludwig Cancer Metastasis Initiative, funded by the Conrad N. Hilton Foundation and the Ludwig Institute for Cancer Research Ltd. PSJ gratefully acknowledges financial support from the Wellcome Trust (078305). The authors are also grateful to Dr Ayad Eddaoudi and Prabhjoat Chana at the flow cytometry core facility, Camelia Botnar Laboratories, Great Ormond Street Hospital for their help and advice on flow cytometry. They would also like to thank Mr Andrew Vaughan at the MRC Laboratory for Molecular Cell Biology & Cell Biology Unit, University College London, for his help in setting up the live imaging experiment.

## Supplementary Material

Additional file 1 **RT-PCR validation**. A table showing the gene name, accession number and primer set used for the two rounds of RT-PCR validation. Click here for file

Additional file 2 **Transcription factors identified to be differentially expressed between normal breast and primary tumours**. Twenty-four transcription factors were identified in the first round of RT-PCR validation to be differentially expressed between normal breast and primary tumours. Two hundred and seventy-five transcription factors identified to be differentially expressed in the DTET underwent the first round of RT-PCR validation using a pool of RNA from normal luminal epithelial cells, a pool of RNA from primary tumours, a pool from F19-negative tumours and RNA from the normal mammary epithelial cell line 226L. Twenty-four transcription factors were confirmed to be deregulated in tumours in the first round of RT-PCR validation. GAPDH or β_2_-microglobulin (β2 M) was used as loading controls. Click here for file

Additional file 3 **NR4A1 is expressed by cells at the edge of the wound in MCF-10A cells ectopically expressing NR4A1**. A wound was scratched in a confluent monolayer of MCF10A-NR4A1 or control cells. The cells were allowed to migrate into the wound overnight. They were then fixed and stained with (A) an anti-NR4A1 antibody and (B) Dapi. Scale bars: 50 μm. Click here for file

Additional file 4 **Time-lapse microscopy**. Control and MCF10A-NR4A1 cells were plated on 35 mm dishes and were allowed to reach confluency. Wounds were scratched using a 200 μl yellow tip and cell migration was monitored over a period of 24 hours, while images of the same fields were taken every 10 minutes. Time-lapse microscopy confirms the reduced rate of migration of MCF10A-NR4A1 cells (Additional file 5) compared with control cells, and shows that wound closure indeed occurs by cell spreading rather than cell proliferation. Click here for file

Additional file 5 **Time-lapse microscopy showing migration of MCF10A-NR4A1 cells**. Control cells and MCF10A-NR4A1 cells were plated on 35 mm dishes and were allowed to reach confluency. Wounds were scratched using a 200 μl yellow tip and cell migration was monitored over a period of 24 hours, while images of the same fields were taken every 10 minutes. Time-lapse microscopy confirms the reduced rate of migration of MCF10A-NR4A1 cells compared with control cells (Additional file 4), and shows that wound closure indeed occurs by cell spreading rather than cell proliferation. Click here for file

Additional file 6 **Flow cytometry analysis of BrdU incorporation in normal and tumour breast cell lines**. Normal and breast cancer cell lines were incubated with BrdU for 1 hour at 37°C. The cells were then stained with an anti-BrdU antibody and analysed by flow cytometry. No significant differences in the rate of entry into S phase were observed between control cells and cells ectopically expressing NR4A1. Click here for file

Additional file 7 **NR4A1 does not induce apoptosis in the normal and breast cancer lines tested**. Growing cells were collected and the Annexin V assay was performed to detect apoptosis. No differences in the apoptotic index between control cells and cells ectopically expressing NR4A1 were observed. Click here for file

## References

[B1] Hu M Polyak K Molecular characterisation of the tumour microenvironment in breast cancer Eur J Cancer 2008 44 2760 2765 10.1016/j.ejca.2008.09.038 19026532 PMC2729518

[B2] Kim JB Stein R O'Hare MJ Tumour-stromal interactions in breast cancer: the role of stroma in tumourigenesis Tumour Biol 2005 26 173 185 10.1159/000086950 16006771

[B3] Da Silva L Clarke C Lakhani SR Demystifying basal-like breast carcinomas J Clin Pathol 2007 60 1328 1332 10.1136/jcp.2006.041731 17496191 PMC2095578

[B4] Lerma E Barnadas A Prat J Triple negative breast carcinomas: similarities and differences with basal like carcinomas Appl Immunohistochem Mol Morphol 2009 17 483 494 10.1097/PAI.0b013e3181a725eb 19620842

[B5] Rakha EA Reis-Filho JS Ellis IO Basal-like breast cancer: a critical review J Clin Oncol 2008 26 2568 2581 10.1200/JCO.2007.13.1748 18487574

[B6] Perou CM Sørlie T Eisen MB van de Rijn M Jeffrey SS Rees CA Pollack JR Ross DT Johnsen H Akslen LA Fluge O Pergamenschikov A Williams C Zhu SX Lønning PE Børresen-Dale AL Brown PO Botstein D Molecular portraits of human breast tumours Nature 2000 406 747 752 10.1038/35021093 10963602

[B7] Sørlie T Perou CM Tibshirani R Aas T Geisler S Johnsen H Hastie T Eisen MB van de Rijn M Jeffrey SS Thorsen T Quist H Matese JC Brown PO Botstein D Eystein Lønning P Børresen-Dale AL Gene expression patterns of breast carcinomas distinguish tumour subclasses with clinical implications Proc Natl Acad Sci USA 2001 98 10869 10874 10.1073/pnas.191367098 11553815 PMC58566

[B8] Ramaswamy S Ross KN Lander ES Golub TR A molecular signature of metastasis in primary solid tumours Nat Genet 2003 33 49 54 10.1038/ng1060 12469122

[B9] van 't Veer LJ Dai H van de Vijver MJ He YD Hart AA Mao M Peterse HL van der Kooy K Marton MJ Witteveen AT Schreiber GJ Kerkhoven RM Roberts C Linsley PS Bernards R Friend SH Gene expression profiling predicts clinical outcome of breast cancer Nature 2002 415 530 536 10.1038/415530a 11823860

[B10] Weigelt B Peterse JL van 't Veer LJ Breast cancer metastasis: markers and models Nat Rev Cancer 2005 5 591 602 10.1038/nrc1670 16056258

[B11] Clarke C Titley J Davies S O'Hare MJ An immunomagnetic separation method using superparamagnetic (MACS) beads for large-scale purification of human mammary luminal and myoepithelial cells Epithelial Cell Biol 1994 3 38 46 7514934

[B12] Grigoriadis A Mackay A Reis-Filho JS Steele D Iseli C Stevenson BJ Jongeneel CV Valgeirsson H Fenwick K Iravani M Leao M Simpson AJ Strausberg RL Jat PS Ashworth A Neville AM O'Hare MJ Establishment of the epithelial-specific transcriptome of normal and malignant human breast cells based on MPSS and array expression data Breast Cancer Res 2006 8 R56 10.1186/bcr1604 17014703 PMC1779497

[B13] Page MJ Amess B Townsend RR Parekh R Herath A Brusten L Zvelebil MJ Stein RC Waterfield MD Davies SC O'Hare MJ Proteomic definition of normal human luminal and myoepithelial breast cells purified from reduction mammoplasties Proc Natl Acad Sci USA 1999 96 12589 12594 10.1073/pnas.96.22.12589 10535966 PMC23001

[B14] O'Hare MJ Bond J Clarke C Takeuchi Y Atherton AJ Berry C Moody J Silver AR Davies DC Alsop AE Neville AM Jat PS Conditional immortalization of freshly isolated human mammary fibroblasts and endothelial cells Proc Natl Acad Sci USA 2001 98 646 651 10.1073/pnas.98.2.646 11209060 PMC14642

[B15] Whitehead RH Bertoncello I Webber LM Pedersen JS A new human breast carcinoma cell line (PMC42) with stem cell characteristics. I. Morphologic characterization J Natl Cancer Inst 1983 70 649 661 6572752

[B16] Git A Spiteri I Blenkiron C Dunning MJ Pole JC Chin SF Wang Y Smith J Livesey FJ Caldas C PMC42, a breast progenitor cancer cell line, has normal-like mRNA and microRNA transcriptomes Breast Cancer Res 2008 10 R54 10.1186/bcr2109 18588681 PMC2481505

[B17] Fuller SJ Sivarajah K Sugden PH ErbB receptors, their ligands, and the consequences of their activation and inhibition in the myocardium J Mol Cell Cardiol 2008 44 831 854 10.1016/j.yjmcc.2008.02.278 18430438

[B18] Irie HY Pearline RV Grueneberg D Hsia M Ravichandran P Kothari N Natesan S Brugge JS Distinct roles of Akt1 and Akt2 in regulating cell migration and epithelial-mesenchymal transition J Cell Biol 2005 171 1023 1034 10.1083/jcb.200505087 16365168 PMC2171329

[B19] Jiang Q Zhou C Bi Z Wan Y EGF-induced cell migration is mediated by ERK and PI3K/AKT pathways in cultured human lens epithelial cells J Ocul Pharmacol Ther 2006 22 93 102 10.1089/jop.2006.22.93 16722795

[B20] Wang Z Yang H Tachado SD Capó-Aponte JE Bildin VN Koziel H Reinach PS Phosphatase-mediated crosstalk control of ERK and p38 MAPK signaling in corneal epithelial cells Invest Ophthalmol Vis Sci 2006 47 5267 5275 10.1167/iovs.06-0642 17122112

[B21] Grigoriadis A Caballero OL Hoek KS da Silva L Chen YT Shin SJ Jungbluth AA Miller LD Clouston D Cebon J Old LJ Lakhani SR Simpson AJ Neville AM CT-X antigen expression in human breast cancer Proc Natl Acad Sci USA 2009 106 13493 13498 10.1073/pnas.0906840106 19651608 PMC2716388

[B22] Bonta PI van Tiel CM Vos M Pols TW van Thienen JV Ferreira V Arkenbout EK Seppen J Spek CA van der Poll T Pannekoek H de Vries CJ Nuclear receptors NR4A1, Nurr1, and NOR-1 expressed in atherosclerotic lesion macrophages reduce lipid loading and inflammatory responses Arterioscler Thromb Vasc Biol 2006 26 2288 2294 10.1161/01.ATV.0000238346.84458.5d 16873729

[B23] Gervais J Soghomonian JJ Richard D Rouillard C Dopamine and serotonin interactions in the modulation of the expression of the immediate-early transcription factor, nerve growth factor-inducible B, in the striatum Neuroscience 1999 91 1045 1054 10.1016/S0306-4522(98)00688-5 10391482

[B24] Honkaniemi J Kononen J Kainu T Pyykönen I Pelto-Huikko M Induction of multiple immediate early genes in rat hypothalamic paraventricular nucleus after stress Brain Res Mol Brain Res 1994 25 234 241 10.1016/0169-328X(94)90158-9 7808222

[B25] Kolluri SK Bruey-Sedano N Cao X Lin B Lin F Han YH Dawson MI Zhang X K Mitogenic effect of orphan receptor TR3 and its regulation by MEKK1 in lung cancer cells Mol Cell Biol 2003 23 8651 8667 10.1128/MCB.23.23.8651-8667.2003 14612408 PMC262666

[B26] Yoo YG Yeo MG Kim DK Park H Lee MO Novel function of orphan nuclear receptor NR4A1 in stabilizing hypoxia-inducible factor-1α J Biol Chem 2004 279 53365 53373 10.1074/jbc.M408554200 15385570

[B27] Philips A Lesage S Gingras R Maira MH Gauthier Y Hugo P Drouin J Novel dimeric NR4A1 signaling mechanism in endocrine and lymphoid cells Mol Cell Biol 1997 17 5946 5951 9315652 10.1128/mcb.17.10.5946 PMC232442

[B28] Lévesque D Rouillard C NR4A1 and retinoid × receptors: crucial factors in dopamine-related neuroadaptation Trends Neurosci 2007 30 22 30 10.1016/j.tins.2006.11.006 17134767 PMC5333988

[B29] Li H Kolluri SK Gu J Dawson MI Cao X Hobbs PD Lin B Chen G Lu J Lin F Xie Z Fontana JA Reed JC Zhang X Cytochrome c release and apoptosis induced by mitochondrial targeting of nuclear orphan receptor TR3 Science 2000 289 1159 1164 10.1126/science.289.5482.1159 10947977

[B30] Martínez-González J Badimon L The NR4A subfamily of nuclear receptors: new early genes regulated by growth factors in vascular cells Cardiovasc Res 2005 65 609 618 10.1016/j.cardiores.2004.10.002 15664387

[B31] Maxwell MA Muscat GE The NR4A subgroup: immediate early response genes with pleiotropic physiological roles Nucl Recept Signal 2006 4 e002 10.1621/nrs.04002 16604165 PMC1402209

[B32] Pols TW Bonta PI de Vries CJ NR4A nuclear orphan receptors: protective in vascular disease? Curr Opin Lipidol 2007 18 515 520 10.1097/MOL.0b013e3282ef77d1 17885421

[B33] Uemura H Chang C Antisense TR3 orphan receptor can increase prostate cancer cell viability with etoposide treatment Endocrinology 1998 139 2329 2534 10.1210/en.139.5.2329 9564841

[B34] Wilson AJ Arango D Mariadason JM Heerdt BG Augenlicht LH TR3/NR4A1 in colon cancer cell apoptosis Cancer Res 2003 63 5401 5407 14500374

[B35] Ye X Wu Q Liu S Lin X Zhang B Wu J Cai J Zhang M Su W Distinct role and functional mode of TR3 and RARalpha in mediating ATRA-induced signalling pathway in breast and gastric cancer cells Int J Biochem Cell Biol 2004 36 98 113 10.1016/S1357-2725(03)00143-2 14592536

[B36] Yu H Kumar SM Fang D Acs G Xu X Nuclear orphan receptor TR3/NR4A1 mediates melanoma cell apoptosis Cancer Biol Ther 2007 6 405 412 10.1158/1535-7163.MCT-07-0268 17297306

[B37] Zeng H Qin L Zhao D Tan X Manseau EJ Van Hoang M Senger DR Brown LF Nagy JA Dvorak HF Orphan nuclear receptor TR3/NR4A1 regulates VEGF-A-induced angiogenesis through its transcriptional activity J Exp Med 2006 203 719 729 10.1084/jem.20051523 16520388 PMC2118245

[B38] Mullican SE Zhang S Konopleva M Ruvolo V Andreeff M Milbrandt J Conneely OM Abrogation of nuclear receptors Nr4a3 and Nr4a1 leads to development of acute myeloid leukemia Nat Med 2007 13 730 735 10.1038/nm1579 17515897

[B39] Alexopoulou AN Multhaupt HA Couchman JR Syndecans in wound healing, inflammation and vascular biology Int J Biochem Cell Biol 2007 39 505 528 10.1016/j.biocel.2006.10.014 17097330

[B40] Streuli CH Akhtar N Signal co-operation between integrins and other receptor systems Biochem J 2009 418 491 506 10.1042/BJ20081948 19228122

