# Supplementary material for: Dissecting the transcriptional networks underlying breast cancer: NR4A1 reduces the migration of normal and breast cancer cell lines
Source: Breast Cancer Res. 2010 Jul 19;12(4):R51. doi: 10.1186/bcr2610 (PMC2949640; doi:10.1186/bcr2610)
Supplement: Additional file 2 — Transcription factors identified to be differentially expressed between normal breast and primary tumours. Twenty-four transcription factors were identified in the first round of RT-PCR validation to be differentially expressed between normal breast and primary tumours. Two hundred and seventy-five transcription factors identified to be differentially expressed in the DTET underwent the first round of RT-PCR validation using a pool of RNA from normal luminal epithelial cells, a pool of RNA from primary tumours, a pool from F19-negative tumours and RNA from the normal mammary epithelial cell line 226L. Twenty-four transcription factors were confirmed to be deregulated in tumours in the first round of RT-PCR validation. GAPDH or β2-microglobulin (β2 M) was used as loading controls. [file bcr2610-S2.PPT]

## Slide 1
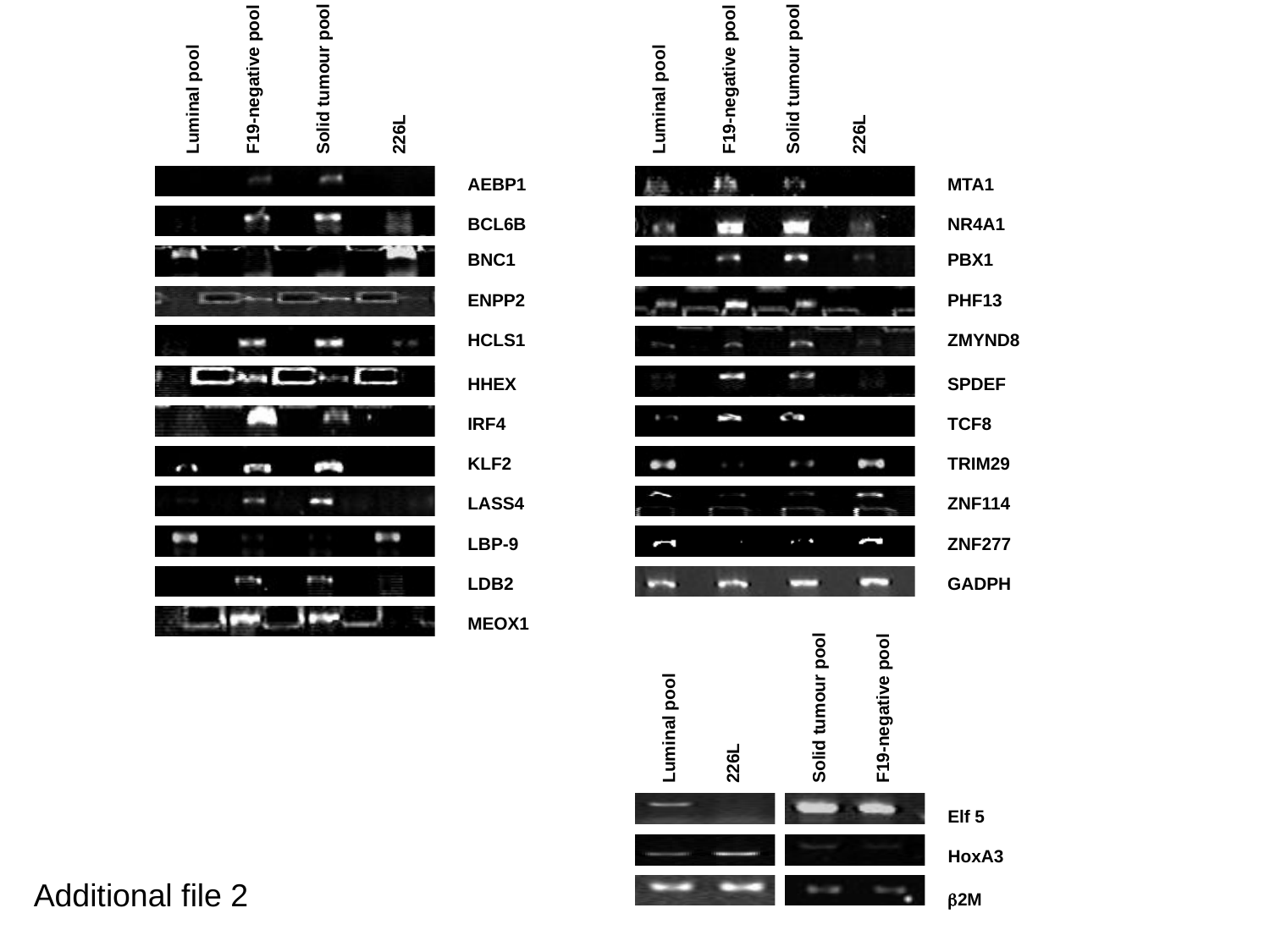

Luminal pool
F19-negative pool
Solid tumour pool
226L
Luminal pool
F19-negative pool
Solid tumour pool
226L
AEBP1
MTA1
BCL6B
NR4A1
BNC1
PBX1
ENPP2
PHF13
HCLS1
ZMYND8
HHEX
SPDEF
IRF4
TCF8
KLF2
TRIM29
LASS4
ZNF114
LBP-9
ZNF277
LDB2
GADPH
Luminal pool
Solid tumour pool
F19-negative pool
226L
MEOX1
Elf 5
HoxA3
Additional file 2
β2M
