# Supplementary figures and images for: Dissecting the transcriptional networks underlying breast cancer: NR4A1 reduces the migration of normal and breast cancer cell lines
Source: Breast Cancer Res. 2010 Jul 19;12(4):R51. doi: 10.1186/bcr2610 (PMC2949640; doi:10.1186/bcr2610)

## Slide 1
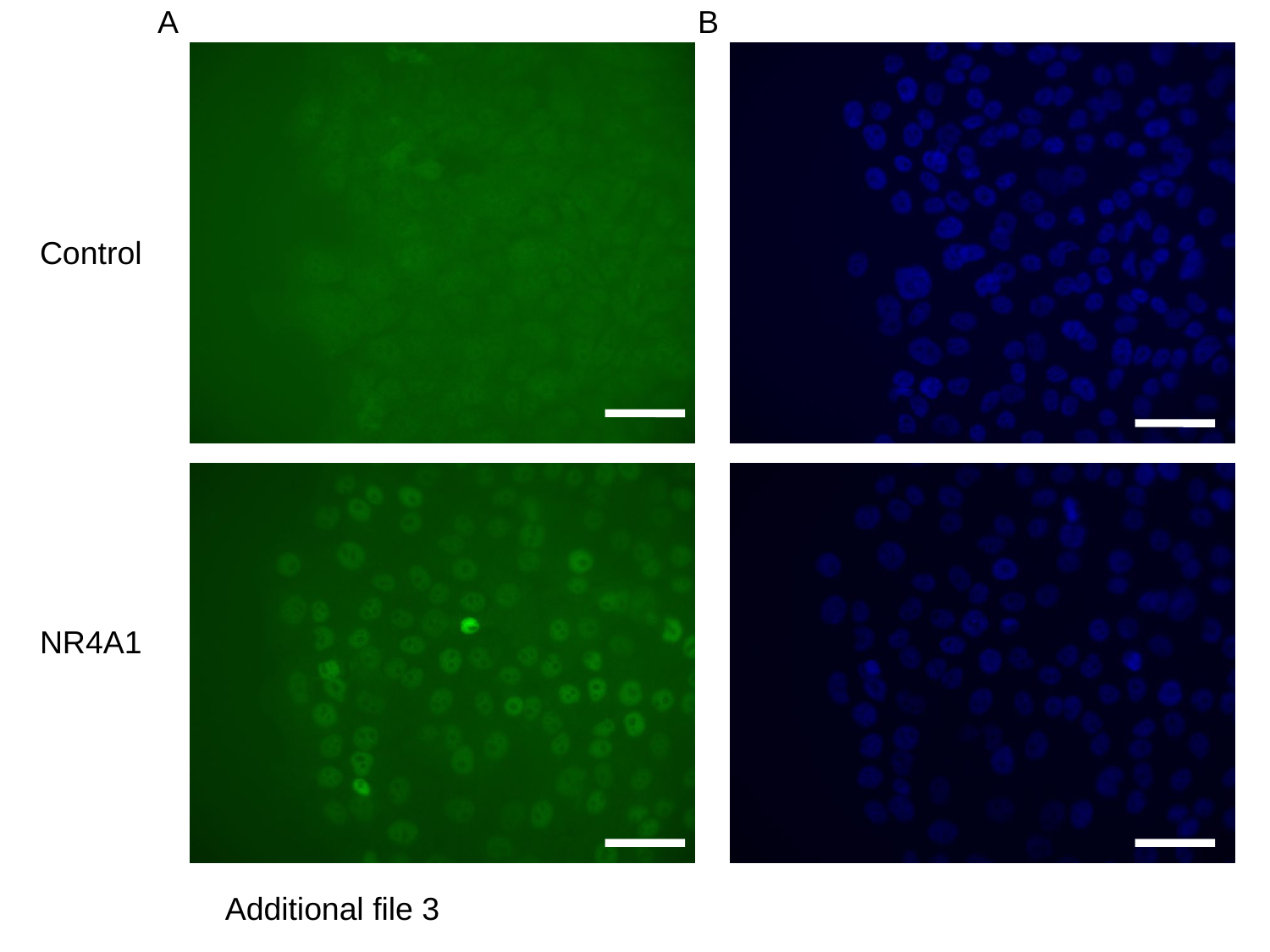

A
B
Control
NR4A1
Additional file 3

Supplement: Additional file 3 — NR4A1 is expressed by cells at the edge of the wound in MCF-10A cells ectopically expressing NR4A1. A wound was scratched in a confluent monolayer of MCF10A-NR4A1 or control cells. The cells were allowed to migrate into the wound overnight. They were then fixed and stained with (A) an anti-NR4A1 antibody and (B) Dapi. Scale bars: 50 μm. [file bcr2610-S3.PPT]

## Slide 1
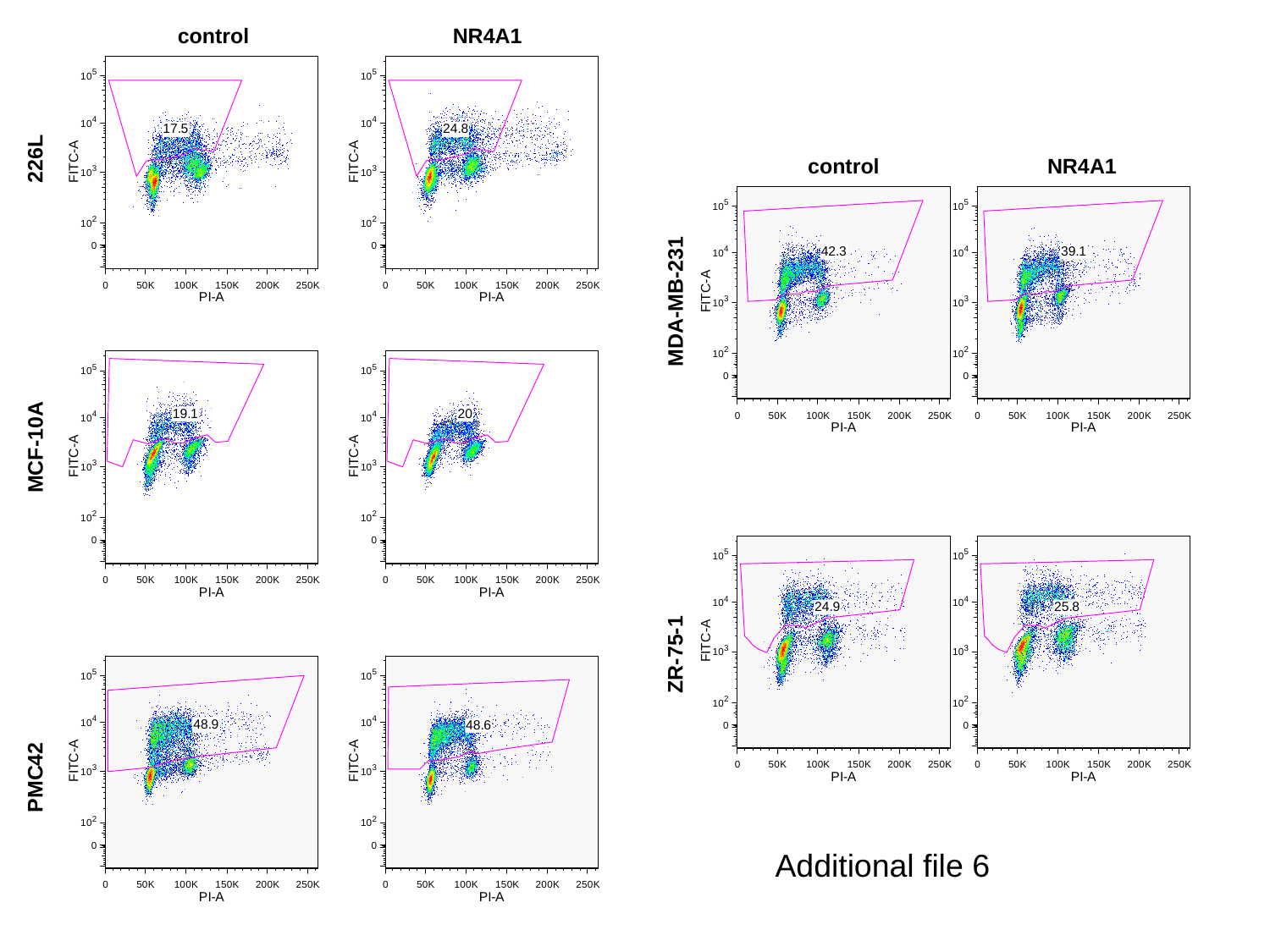

control
NR4A1
226L
control
NR4A1
MDA-MB-231
MCF-10A
ZR-75-1
PMC42
Additional file 6

Supplement: Additional file 6 — Flow cytometry analysis of BrdU incorporation in normal and tumour breast cell lines. Normal and breast cancer cell lines were incubated with BrdU for 1 hour at 37°C. The cells were then stained with an anti-BrdU antibody and analysed by flow cytometry. No significant differences in the rate of entry into S phase were observed between control cells and cells ectopically expressing NR4A1. [file bcr2610-S6.PPT]

## Slide 1
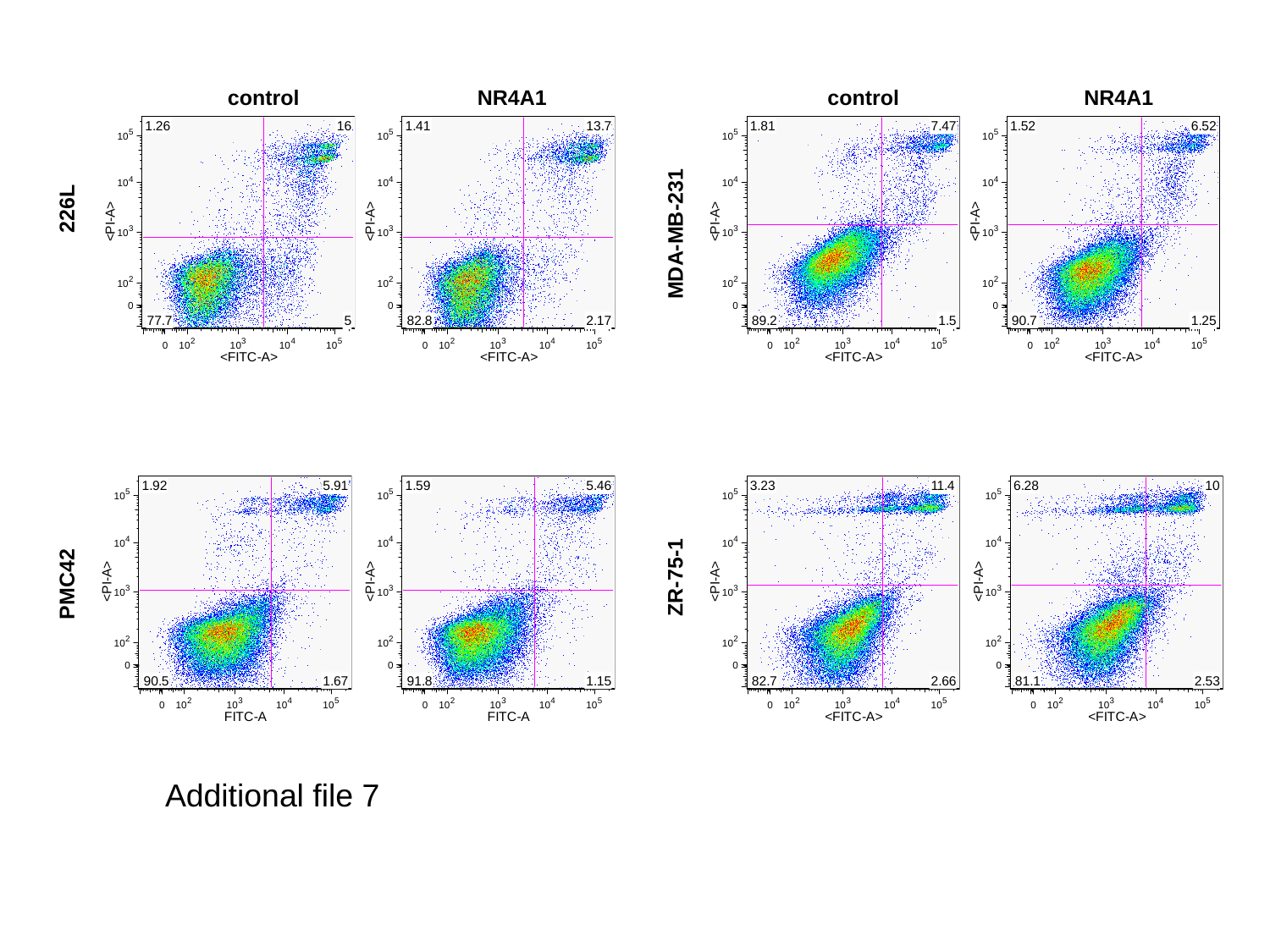

control
NR4A1
control
NR4A1
226L
MDA-MB-231
ZR-75-1
PMC42
Additional file 7

Supplement: Additional file 7 — NR4A1 does not induce apoptosis in the normal and breast cancer lines tested. Growing cells were collected and the Annexin V assay was performed to detect apoptosis. No differences in the apoptotic index between control cells and cells ectopically expressing NR4A1 were observed. [file bcr2610-S7.PPT]
